# 2,5-Bis(bromo­meth­yl)biphen­yl

**DOI:** 10.1107/S1600536809018066

**Published:** 2009-05-20

**Authors:** Piotr Kuś, Aleksander Zemanek, Peter G. Jones

**Affiliations:** a Department of Chemistry, University of Silesia, 9 Szkolna Street, 40-006 Katowice, Poland; b Institut für Anorganische und Analytische Chemie, Technische Universität Braunschweig, Postfach 3329, 38023 Braunschweig, Germany

## Abstract

In the title compound, C_14_H_12_Br_2_, the Br atoms lie on opposite sides of their ring plane. The biphenyl inter­planar angle is 53.52 (8)°. The packing is characterized by several H⋯Br contacts to each Br atom, but at long distances of 3.07–3.43 Å.

## Related literature

For the structures of bromo­methyl-substituted aromatic ring systems, see: Jones & Kuś (2005 ▶, 2007 ▶); Jones *et al.* (2007 ▶). For the synthesis, see: Czuchajowski & Zemanek (1990 ▶); For a related structure with a similar conformation, see: Obrey *et al.* (2002 ▶). For the phenomenon of tertiary contacts, see: Du Mont *et al.* (2008 ▶);
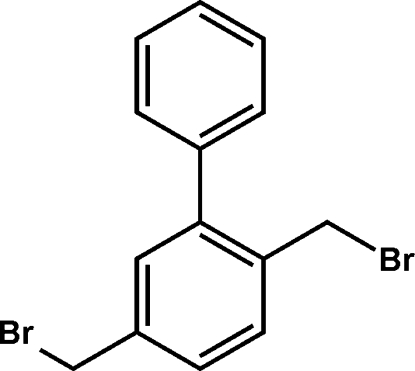


## Experimental

### 

#### Crystal data

C_14_H_12_Br_2_
*M* *_r_* = 340.06
Monoclinic, 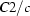

*a* = 33.084 (4) Å
*b* = 4.3354 (6) Å
*c* = 18.017 (2) Å
β = 103.702 (4)°
*V* = 2510.7 (5) Å^3^
*Z* = 8
Mo *K*α radiation
μ = 6.43 mm^−1^
*T* = 133 K
0.25 × 0.10 × 0.10 mm

#### Data collection

Bruker SMART 1000 CCD area-detector diffractometer
Absorption correction: multi-scan (*SADABS*; Bruker, 1998 ▶) *T*_min_ = 0.316, *T*_max_ = 0.566 (expected range = 0.294–0.526)
18269 measured reflections
3120 independent reflections
2518 reflections with *I* > 2σ(*I*)
*R*_int_ = 0.040

#### Refinement

*R*[*F* ^2^ > 2σ(*F* ^2^)] = 0.031
*wR*(*F* ^2^) = 0.085
*S* = 1.05
3120 reflections
145 parameters
H-atom parameters constrained
Δρ_max_ = 1.00 e Å^−3^
Δρ_min_ = −1.00 e Å^−3^

### 

Data collection: *SMART* (Bruker, 1998 ▶); cell refinement: *SAINT* (Bruker, 1998 ▶); data reduction: *SAINT*; program(s) used to solve structure: *SHELXS97* (Sheldrick, 2008 ▶); program(s) used to refine structure: *SHELXL97* (Sheldrick, 2008 ▶); molecular graphics: *XP* (Siemens, 1994 ▶); software used to prepare material for publication: *SHELXL97*.

## Supplementary Material

Crystal structure: contains datablocks I, global. DOI: 10.1107/S1600536809018066/bt2951sup1.cif

Structure factors: contains datablocks I. DOI: 10.1107/S1600536809018066/bt2951Isup2.hkl

Additional supplementary materials:  crystallographic information; 3D view; checkCIF report

## Figures and Tables

**Table 1:** H⋯Br contacts (Å, °)

*D*—H⋯*A*	*D*—H	H⋯*A*	*D*⋯*A*	*D*—H⋯*A*
C7—H7*B*⋯Br1^i^	0.99	3.23	3.773 (3)	116
C12—H12⋯Br1^ii^	0.95	3.07	3.782 (3)	133
C13—H13⋯Br1^ii^	0.95	3.37	3.931 (3)	120
C13—H13⋯Br1^iii^	0.95	3.24	3.634 (3)	107
C14—H14⋯Br1^iv^	0.95	3.37	3.971 (3)	123
C14—H14⋯Br1^v^	0.95	3.43	4.326 (3)	157
C4—H4⋯Br2^vi^	0.95	3.37	4.260 (3)	156
C4—H4⋯Br2^vii^	0.95	3.26	3.845 (3)	122
C6—H6⋯Br2^viii^	0.95	3.20	4.124 (3)	166
C8—H8*B*⋯Br2^ix^	0.99	3.29	3.746 (3)	110
C8—H8*A*⋯Br2^ix^	0.99	3.43	3.746 (3)	101
C15—H15⋯Br2^x^	0.95	3.27	3.913 (3)	127
C16—H16⋯Br2^viii^	0.95	3.41	3.918 (3)	116
C16—H16⋯Br2^x^	0.95	3.24	3.898 (3)	128

Symmetry codes: (i) 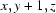; (ii) 
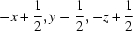
; (iii) 
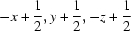
; (iv) 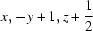; (v) 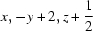; (vi) 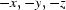; (vii) 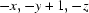; (viii) 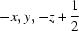; (ix) 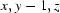; (x) 
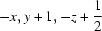
.

**Fig. 1.**
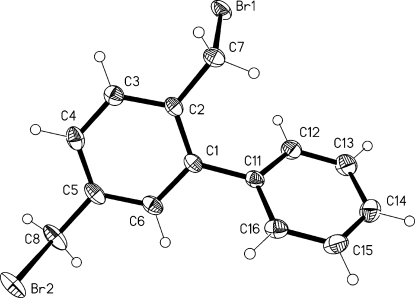
The title compound in the crystal. Displacement ellipsoids represent 50% probability levels.

**Fig. 2.**
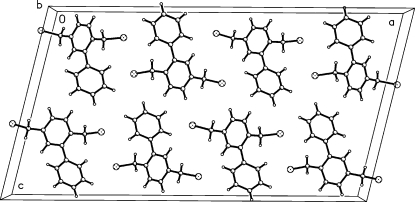
Packing diagram of the title compound viewed parallel to the short b axis.

## supplementary crystallographic information

### Comment

We are interested in the structures of bromomethyl-substituted aromatic ring systems, compounds that are often used as synthestic intermediates; *e.g.* various bromomethylbenzenes (Jones & Kuś, 2007), 2,2"- and 2',5'- bis(bromomethyl)-*p*-terphenyl (Jones & Kuś, 2005; Jones *et al.*, 2007). The packing patterns are often characterized by secondary interactions such as C—H···Br, Br···Br and C—H···π.

As a part of the synthesis of phenyl derivatives of [2.2]paracyclophane (Czuchajowski & Zemanek 1990), 2,5-di(bromomethyl)biphenyl (1) was obtained by bromination of 2,5-dimethylbiphenyl. Here we present its structure (Fig. 1).

Bond lengths and angles may be regarded as normal (*e.g.* the single bond between the rings is 1.486 (4) Å; ring angles at the substituted atoms C1, C2, C5 are all about 1° less than the ideal 120°). The interplanar angle is 53.52 (8)°. The bromomethyl groups adopt an *anti*-conformation whereby Br1 and Br2 lie out of their ring plane by 1.680 (4) and -1.736 (4) Å; associated torsion angles are C3—C2—C7—Br1 - 77.2 (3) and C4—C5—C8—Br2 - 87.9 (3)°. A similar conformation was observed in 2,6-di(bromomethyl)biphenyl (Obrey *et al.*, 2002), whereas 2',5'-di(bromomethyl)-*p*-terphenyl adopts a *syn*-conformation (Jones *et al.* 2007).

The packing (Fig. 2) appears at first sight to be characterized by an almost total lack of secondary contacts. The shortest H···Br contact is H12···Br1 3.07 Å (operator 0.5 - *x*,-1/2 + *y*,0.5 - *z*) and there are no other H···Br < 3.19 Å; there are no Br···Br contacts < 4.2 Å and no H···π contacts < 2.95 Å (and these with very narrow angles). Both bromine atoms however are situated in a pocket surrounded by several H atoms; Br1 by six H at distances of 3.07–3.43, Br2 by eight H from 3.20–3.43 Å. This corresponds to the phenomenon of *tertiary contacts* as postulated by Du Mont *et al.* (2008).

### Experimental

The title compound was obtained from 2,5-dimethylbiphenyl according to the method of Czuchajowski & Zemanek (1990). The analytical and spectroscopic data are consistent with the literature. Single crystals were grown by slow evaporation of a hexane solution. NMR data for (1): ^1^H NMR (CDCl_3_, 400 MHz): δ 7.52 (d, 1H), 7.50–7.39 (m, 6H), 7.29 (d, 1H), 4.50 (s, 2H), 4.44(s, 2H); ^13^C NMR (100 MHz): δ 142.54, 139.57, 138.07, 135.46, 131.53, 131.03, 128.94, 128.61, 128.44, 127.77, 32.75, 31.58.

### Refinement

H atoms were included at calculated positions and refined using a riding model, with fixed C—H bond lengths of 0.95 Å (CH, aromatic) or 0.99 Å (CH_2_) Å; *U*_iso_(H) values were fixed at 1.2*U*_eq_ of the parent C atom. Largest difference peaks of ±1.0 e Å^-3^ near the bromine atoms may be attributed to residual absorption errors.

### Crystal data

**Table:**

C_14_H_12_Br_2_	*F*(000) = 1328
*M**_r_* = 340.06	*D*_x_ = 1.799 Mg m^−^^3^
Monoclinic, *C*2/*c*	Mo *K*α radiation, λ = 0.71073 Å
*a* = 33.084 (4) Å	Cell parameters from 6730 reflections
*b* = 4.3354 (6) Å	θ = 2.3–28.8°
*c* = 18.017 (2) Å	µ = 6.43 mm^−^^1^
β = 103.702 (4)°	*T* = 133 K
*V* = 2510.7 (5) Å^3^	Prism, colourless
*Z* = 8	0.25 × 0.10 × 0.10 mm

### Data collection

**Table:**

Bruker SMART 1000 CCD area-detector diffractometer	3120 independent reflections
Radiation source: fine-focus sealed tube	2518 reflections with *I* > 2σ(*I*)
graphite	*R*_int_ = 0.040
Detector resolution: 8.192 pixels mm^-1^	θ_max_ = 28.3°, θ_min_ = 1.3°
φ and ω scans	*h* = −44→44
Absorption correction: multi-scan (*SADABS*; Bruker, 1998)	*k* = −5→5
*T*_min_ = 0.316, *T*_max_ = 0.566	*l* = −24→24
18269 measured reflections	

### Refinement

**Table:**

Refinement on *F*^2^	Primary atom site location: structure-invariant direct methods
Least-squares matrix: full	Secondary atom site location: difference Fourier map
*R*[*F*^2^ > 2σ(*F*^2^)] = 0.031	Hydrogen site location: inferred from neighbouring sites
*wR*(*F*^2^) = 0.085	H-atom parameters constrained
*S* = 1.05	*w* = 1/[σ^2^(*F*_o_^2^) + (0.0515*P*)^2^ + 1.9201*P*] where *P* = (*F*_o_^2^ + 2*F*_c_^2^)/3
3120 reflections	(Δ/σ)_max_ = 0.001
145 parameters	Δρ_max_ = 1.00 e Å^−^^3^
0 restraints	Δρ_min_ = −1.00 e Å^−^^3^

### Special details

**Table:**

Geometry. All e.s.d.'s (except the e.s.d. in the dihedral angle between two l.s. planes) are estimated using the full covariance matrix. The cell e.s.d.'s are taken into account individually in the estimation of e.s.d.'s in distances, angles and torsion angles; correlations between e.s.d.'s in cell parameters are only used when they are defined by crystal symmetry. An approximate (isotropic) treatment of cell e.s.d.'s is used for estimating e.s.d.'s involving l.s. planes.
Refinement. Refinement of *F*^2^ against ALL reflections. The weighted *R*-factor *wR* and goodness of fit *S* are based on *F*^2^, conventional *R*-factors *R* are based on *F*, with *F* set to zero for negative *F*^2^. The threshold expression of *F*^2^ > σ(*F*^2^) is used only for calculating *R*-factors(gt) *etc*. and is not relevant to the choice of reflections for refinement. *R*-factors based on *F*^2^ are statistically about twice as large as those based on *F*, and *R*- factors based on ALL data will be even larger.

### Fractional atomic coordinates and isotropic or equivalent isotropic displacement parameters (Å^2^)

**Table:**

	*x*	*y*	*z*	*U*_iso_*/*U*_eq_	
Br1	0.219743 (8)	0.60712 (7)	0.152339 (16)	0.02600 (10)	
Br2	−0.025878 (8)	0.25836 (8)	0.108852 (19)	0.03229 (11)	
C1	0.11964 (8)	0.5306 (6)	0.22477 (14)	0.0176 (5)	
C2	0.13425 (8)	0.6193 (6)	0.16080 (15)	0.0179 (5)	
C3	0.11266 (8)	0.5205 (7)	0.08827 (15)	0.0209 (5)	
H3	0.1219	0.5856	0.0447	0.025*	
C4	0.07831 (9)	0.3310 (7)	0.07839 (17)	0.0244 (6)	
H4	0.0647	0.2621	0.0287	0.029*	
C5	0.06355 (8)	0.2407 (6)	0.14127 (17)	0.0222 (6)	
C6	0.08387 (8)	0.3445 (7)	0.21324 (16)	0.0209 (6)	
H6	0.0733	0.2883	0.2560	0.025*	
C7	0.17006 (8)	0.8325 (7)	0.16619 (16)	0.0211 (6)	
H7A	0.1765	0.9347	0.2168	0.025*	
H7B	0.1625	0.9942	0.1265	0.025*	
C8	0.02710 (8)	0.0280 (7)	0.13151 (19)	0.0304 (7)	
H8A	0.0294	−0.0937	0.1788	0.037*	
H8B	0.0273	−0.1176	0.0893	0.037*	
C11	0.14006 (8)	0.6257 (7)	0.30405 (14)	0.0187 (5)	
C12	0.18247 (9)	0.5758 (7)	0.33539 (16)	0.0247 (6)	
H12	0.1989	0.4783	0.3056	0.030*	
C13	0.20058 (9)	0.6669 (8)	0.40922 (17)	0.0300 (7)	
H13	0.2293	0.6288	0.4300	0.036*	
C14	0.17707 (10)	0.8145 (8)	0.45354 (17)	0.0306 (7)	
H14	0.1898	0.8822	0.5038	0.037*	
C15	0.13489 (9)	0.8614 (8)	0.42342 (16)	0.0279 (7)	
H15	0.1186	0.9615	0.4532	0.033*	
C16	0.11642 (9)	0.7626 (7)	0.34998 (16)	0.0225 (6)	
H16	0.0873	0.7883	0.3306	0.027*	

### Atomic displacement parameters (Å^2^)

**Table:**

	*U*^11^	*U*^22^	*U*^33^	*U*^12^	*U*^13^	*U*^23^
Br1	0.01373 (14)	0.03068 (18)	0.03439 (16)	−0.00368 (11)	0.00730 (10)	−0.00446 (13)
Br2	0.01203 (15)	0.0309 (2)	0.0514 (2)	−0.00186 (11)	0.00244 (12)	0.00305 (14)
C1	0.0114 (11)	0.0171 (13)	0.0231 (12)	0.0036 (10)	0.0018 (9)	0.0018 (11)
C2	0.0130 (11)	0.0162 (13)	0.0236 (12)	0.0016 (10)	0.0027 (9)	0.0000 (11)
C3	0.0177 (12)	0.0208 (14)	0.0234 (12)	0.0028 (11)	0.0036 (10)	0.0026 (11)
C4	0.0165 (13)	0.0260 (15)	0.0273 (14)	0.0032 (11)	−0.0013 (10)	−0.0023 (12)
C5	0.0120 (12)	0.0158 (14)	0.0371 (15)	0.0029 (10)	0.0025 (11)	−0.0001 (12)
C6	0.0135 (12)	0.0208 (14)	0.0278 (13)	0.0025 (10)	0.0033 (10)	0.0051 (11)
C7	0.0174 (13)	0.0201 (14)	0.0264 (13)	0.0016 (11)	0.0064 (10)	0.0013 (11)
C8	0.0137 (13)	0.0213 (15)	0.0528 (19)	0.0011 (12)	0.0009 (12)	0.0013 (14)
C11	0.0139 (12)	0.0221 (14)	0.0194 (12)	0.0007 (10)	0.0027 (9)	0.0045 (11)
C12	0.0162 (13)	0.0326 (17)	0.0252 (13)	0.0037 (12)	0.0049 (10)	−0.0003 (12)
C13	0.0156 (13)	0.045 (2)	0.0266 (14)	0.0033 (13)	−0.0003 (11)	0.0033 (14)
C14	0.0276 (16)	0.0415 (19)	0.0209 (13)	−0.0006 (14)	0.0018 (11)	0.0002 (13)
C15	0.0260 (15)	0.0366 (18)	0.0233 (13)	0.0043 (13)	0.0104 (11)	0.0026 (13)
C16	0.0174 (13)	0.0252 (16)	0.0254 (13)	0.0055 (11)	0.0059 (10)	0.0057 (12)

### Geometric parameters (Å, °)

**Table:**

Br1—C7	1.978 (3)	C14—C15	1.387 (4)
Br2—C8	1.974 (3)	C15—C16	1.387 (4)
C1—C2	1.405 (4)	C3—H3	0.9500
C1—C6	1.407 (4)	C4—H4	0.9500
C1—C11	1.486 (4)	C6—H6	0.9500
C2—C3	1.400 (4)	C7—H7A	0.9900
C2—C7	1.487 (4)	C7—H7B	0.9900
C3—C4	1.379 (4)	C8—H8A	0.9900
C4—C5	1.392 (4)	C8—H8B	0.9900
C5—C6	1.387 (4)	C12—H12	0.9500
C5—C8	1.495 (4)	C13—H13	0.9500
C11—C16	1.398 (4)	C14—H14	0.9500
C11—C12	1.400 (4)	C15—H15	0.9500
C12—C13	1.381 (4)	C16—H16	0.9500
C13—C14	1.395 (4)		
			
C2—C1—C6	118.5 (2)	C3—C4—H4	120.0
C2—C1—C11	123.1 (2)	C5—C4—H4	120.0
C6—C1—C11	118.4 (2)	C5—C6—H6	119.0
C3—C2—C1	118.9 (2)	C1—C6—H6	119.0
C3—C2—C7	118.3 (2)	C2—C7—H7A	109.4
C1—C2—C7	122.7 (2)	Br1—C7—H7A	109.4
C4—C3—C2	121.7 (3)	C2—C7—H7B	109.4
C3—C4—C5	120.0 (3)	Br1—C7—H7B	109.4
C6—C5—C4	119.0 (3)	H7A—C7—H7B	108.0
C6—C5—C8	120.6 (3)	C5—C8—H8A	109.4
C4—C5—C8	120.4 (3)	Br2—C8—H8A	109.4
C5—C6—C1	121.9 (3)	C5—C8—H8B	109.4
C2—C7—Br1	111.0 (2)	Br2—C8—H8B	109.4
C5—C8—Br2	111.4 (2)	H8A—C8—H8B	108.0
C16—C11—C12	118.3 (3)	C13—C12—H12	119.7
C16—C11—C1	119.7 (2)	C11—C12—H12	119.7
C12—C11—C1	121.9 (2)	C12—C13—H13	119.7
C13—C12—C11	120.6 (3)	C14—C13—H13	119.7
C12—C13—C14	120.6 (3)	C15—C14—H14	120.4
C15—C14—C13	119.3 (3)	C13—C14—H14	120.4
C14—C15—C16	120.2 (3)	C14—C15—H15	119.9
C15—C16—C11	120.9 (3)	C16—C15—H15	119.9
C4—C3—H3	119.1	C15—C16—H16	119.5
C2—C3—H3	119.1	C11—C16—H16	119.5
			
C6—C1—C2—C3	0.2 (4)	C6—C5—C8—Br2	93.8 (3)
C11—C1—C2—C3	−179.4 (3)	C4—C5—C8—Br2	−87.9 (3)
C6—C1—C2—C7	175.8 (3)	C2—C1—C11—C16	128.0 (3)
C11—C1—C2—C7	−3.8 (4)	C6—C1—C11—C16	−51.6 (4)
C1—C2—C3—C4	−2.1 (4)	C2—C1—C11—C12	−53.6 (4)
C7—C2—C3—C4	−177.8 (3)	C6—C1—C11—C12	126.8 (3)
C2—C3—C4—C5	1.9 (4)	C16—C11—C12—C13	−1.6 (4)
C3—C4—C5—C6	0.1 (4)	C1—C11—C12—C13	179.9 (3)
C3—C4—C5—C8	−178.2 (3)	C11—C12—C13—C14	−1.0 (5)
C4—C5—C6—C1	−1.9 (4)	C12—C13—C14—C15	1.8 (5)
C8—C5—C6—C1	176.3 (3)	C13—C14—C15—C16	−0.1 (5)
C2—C1—C6—C5	1.8 (4)	C14—C15—C16—C11	−2.5 (5)
C11—C1—C6—C5	−178.6 (3)	C12—C11—C16—C15	3.3 (4)
C3—C2—C7—Br1	−77.2 (3)	C1—C11—C16—C15	−178.2 (3)
C1—C2—C7—Br1	107.2 (3)		

### Hydrogen-bond geometry (Å, °)

**Table:**

*D*—H···*A*	*D*—H	H···*A*	*D*···*A*	*D*—H···*A*
C7—H7B···Br1^i^	0.99	3.23	3.773 (3)	116
C12—H12···Br1^ii^	0.95	3.07	3.782 (3)	133
C13—H13···Br1^ii^	0.95	3.37	3.931 (3)	120
C13—H13···Br1^iii^	0.95	3.24	3.634 (3)	107
C14—H14···Br1^iv^	0.95	3.37	3.971 (3)	123
C14—H14···Br1^v^	0.95	3.43	4.326 (3)	157
C4—H4···Br2^vi^	0.95	3.37	4.260 (3)	156
C4—H4···Br2^vii^	0.95	3.26	3.845 (3)	122
C6—H6···Br2^viii^	0.95	3.20	4.124 (3)	166
C8—H8B···Br2^ix^	0.99	3.29	3.746 (3)	110
C8—H8A···Br2^ix^	0.99	3.43	3.746 (3)	101
C15—H15···Br2^x^	0.95	3.27	3.913 (3)	127
C16—H16···Br2^viii^	0.95	3.41	3.918 (3)	116
C16—H16···Br2^x^	0.95	3.24	3.898 (3)	128

Symmetry codes: (i) *x*, *y*+1, *z*; (ii) −*x*+1/2, *y*−1/2, −*z*+1/2; (iii) −*x*+1/2, *y*+1/2, −*z*+1/2; (iv) *x*, −*y*+1, *z*+1/2; (v) *x*, −*y*+2, *z*+1/2; (vi) −*x*, −*y*, −*z*; (vii) −*x*, −*y*+1, −*z*; (viii) −*x*, *y*, −*z*+1/2; (ix) *x*, *y*−1, *z*; (x) −*x*, *y*+1, −*z*+1/2.
